# Inferior Vena Cava Duplication: Incidental Case in a Young Woman

**DOI:** 10.1155/2016/3071873

**Published:** 2016-04-27

**Authors:** Danilo Coco, Sara Cecchini, Silvana Leanza, Massimo Viola, Stefano Ricci, Roberto Campagnacci

**Affiliations:** ^1^Department of Surgery, Card. G. Panico Hospital of Tricase, 73039 Tricase, Italy; ^2^Department of Radiology, INRCA Hospital of Ancona, 60125 Ancona, Italy; ^3^Department of Surgery, C. Urbani Hospital of Jesi, 60035 Jesi, Italy

## Abstract

A case of a double inferior vena cava (IVC) with retroaortic left renal vein, azygos continuation of the IVC, and presence of the hepatic portion of the IVC drained into the right renal vein is reported and the embryologic, clinical, and radiological significance is discussed. The diagnosis is suggested by multidetector computed tomography (MDCT), which reveals the aberrant vascular structures. Awareness of different congenital anomalies of IVC is necessary for radiologists to avoid diagnostic pitfalls and they should be remembered because they can influence several surgical interventions and endovascular procedures.

## 1. Introduction

The first case of inferior vena cava duplication (IVCD) was described in 1916 in a male subject dissected during an autopsy by Lucas in London [1]. As reported in the literature, the incidence of IVCD is 1.5% (range 0.2%–3%), with intraoperative findings between 0.2% and 0.6% [2–4]. Although congenital anomalies of the IVC cases are usually clinically silent and often detected incidentally by imaging, these venous anomalies may have important relevance during retroperitoneal surgery and venous interventional radiologic procedures [5]. We present one case of a double inferior vena cava (IVC) with retroaortic left renal vein, azygos continuation of the IVC, and presence of the hepatic portion of the IVC drained into the right renal vein, demonstrated by a multidetector computed tomography (MDCT).

## 2. Case Report

A 42-year-old woman with abdominal pain in right quadrant was referred to our radiology department for abdominal multidetector computed tomography (MDCT) examination. Abdominal MDCT (BrightSpeed Elite Select, GE Medical System) was performed before and after administration of intravenous contrast medium (150 mL of nonionic contrast material containing 320 mg of iopromide per milliliter) with 16 × 1.25 mm slice collimation, 1.25 mm slice thicknesses. 2D and 3D postprocessing images, such as multiplanar reformation (MPR) and volume rendering (VR) images, were obtained on the workstation for better visualization of vascular structures.

**Figure 1:**
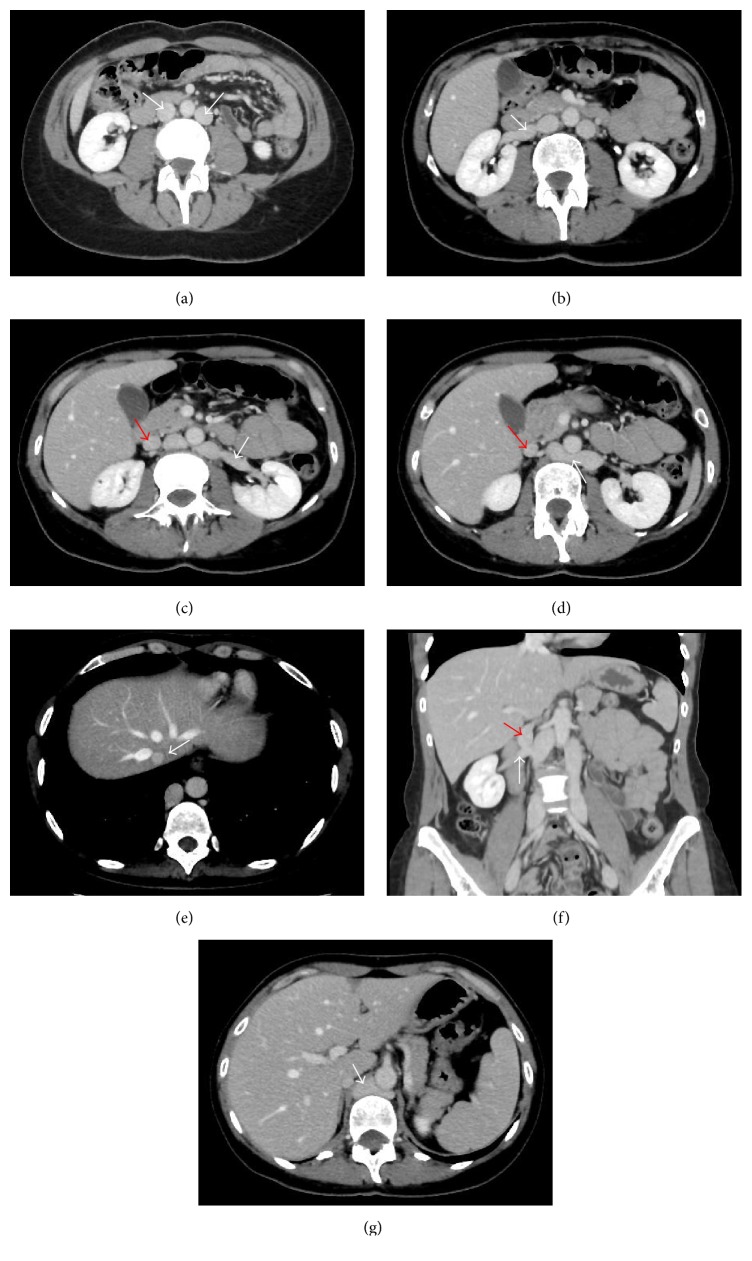
(a) CT axial images that presented from caudal to cranial sections show double IVC (arrows) below the renal veins. (b) The right IVC receives the right renal vein (arrow). (c) The left IVC receives the left renal vein (white arrow); presence of the hepatic portion (red arrow) of the IVC in the CT section before draining into the right renal vein. (d) The left renal vein (white arrow) crosses posterior to the aorta to join the right IVC; continuation of hepatic segment (red arrow). (e) The hepatic veins drain into a hypoplastic hepatic segment (arrow) of the IVC. (f) Coronal MPR image shows the hypoplastic hepatic segment (red arrow) draining into the right renal vein (white arrow), at the confluence with the right IVC. (g) The right IVC continues cephalad as the azygos vein (arrow) within the retrocrural space.

MDCT showed double IVC below the renal veins. Both IVC were formed from the respective common iliac veins and ran upwards bilaterally to the abdominal aorta as far as the renal veins. The left IVC terminated on the left renal vein. The left renal vein crossed posterior to the aorta to join the right IVC. The right IVC, once receiving the left and right renal veins, ran upwards as a single vein continuing superiorly as the azygos vein within the retrocrural space. The azygos vein drained into superior vena cava in the right paratracheal space. The hepatic veins drained into a stump of the hepatic segment of the IVC, which opened cranially into the right atrium and caudally drained into the right renal vein, at the confluence with right IVC (Figures 1(a)–1(g)).

## 3. Discussion

The normal IVC is composed of four segments: hepatic, suprarenal, renal, and infrarenal. It derives from a complex embryogenic process beginning at the sixth week of gestation and involving three pairs of primitive veins (posterior cardinal, subcardinal, and supracardinal veins) that appear and regress anastomozing in the final IVC. In particular, the postcardinal veins appear and remain in the pelvis as the common iliac veins, the right supracardinal vein persists to form the infrarenal IVC, and the right subcardinal vein persists to develop into the suprarenal segment by formation of the subcardinal-hepatic anastomosis while the left subcardinal vein and the left supracardinal vein regress completely [2, 4, 6]. The renal segment develops from the anastomosis between the subcardinal and supracardinal veins while the hepatic segment derives from the right vitelline vein [6, 7].

An alteration of one step of this process determines at least 14 different anatomic anomalies of the IVC and many classification systems have been proposed to group these variants. As reported by Bass, major anomalies are double IVC (with a prevalence of 0.2–3%), left IVC (0.2–0.5%), retroaortic left renal vein (2.1%), circumaortic left renal vein (8.7%), and absence of the hepatic segment of the IVC with azygos continuation of the IVC (0.6% of cases).

The duplication of IVC results from persistence of the right and the left supracardinal veins [8, 9]. Recognition of IVCD is clinically relevant during retroperitoneal surgery or vascular interventional procedures in order to avoid recurrent pulmonary embolism following placement of an IVC filter [10].

The retroaortic left renal vein results from regression of the anterior intersubcardinal (sopra-subcardinal) anastomosis and persistence of the posterior intersupracardinal anastomosis so that a single renal vein passes posterior to the aorta. The clinical significance is in preoperative planning prior to nephrectomy. The presence of retroaortic left renal vein may also cause clinical symptoms such as abdominal pain and hematuria.

Azygos continuation of the IVC has also been named absence of the hepatic segment of the IVC with azygos continuation. The embryologic basis is the failure to form the right subcardinal, hepatic anastomosis.

It is important to be aware of anatomy of this variation in planning cardiopulmonary bypass, in catheterizing the heart, and in the differential diagnosis of right-sided paratracheal mass or retrocrural and paravertebral lymphadenopathy [11–15].

As described in the literature, in some case, more than one variation can coexist as double IVC with retroaortic right renal vein and hemiazygos continuation of the IVC, duplication of the inferior vena cava with azygos continuation, and retroaortic left renal vein and iliac vein variations. Particularly Bass et al. describe the possibility of double IVC with retroaortic left renal vein and azygos continuation of the IVC [6, 16].

The case we describe has a complex combination of these venous variations, which is extremely rare to find together in a patient.

**Figure 2:**
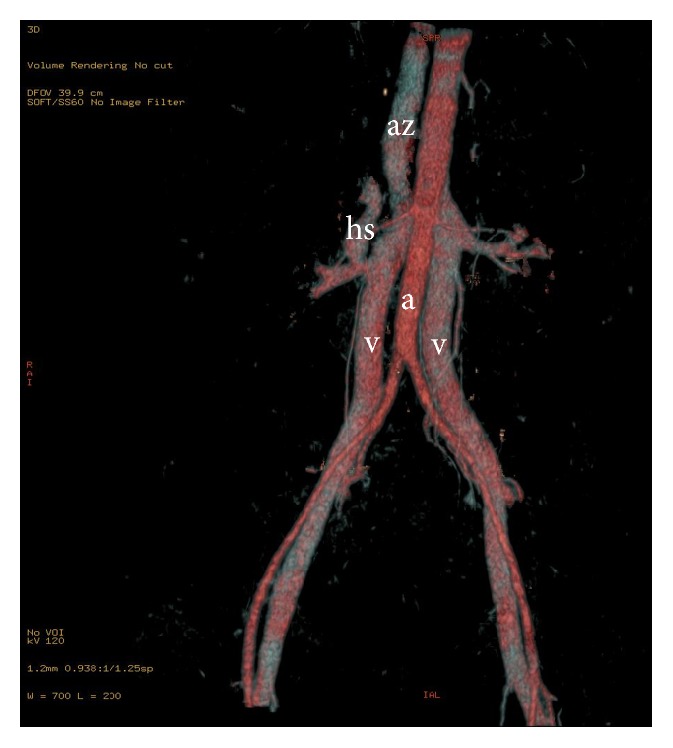
VR image coronal reconstruction shows double IVC (v), azygos continuation of the right IVC (az), and the hypoplastic hepatic segment (hs) of the IVC draining into the right IVC and the aorta (a).

**Figure 3:**
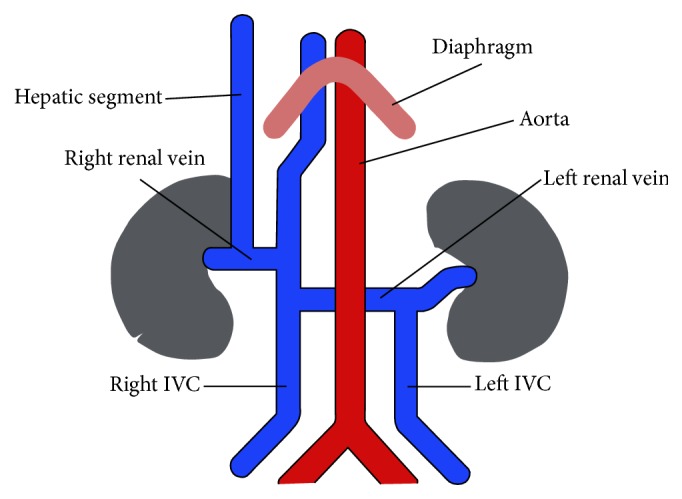
Schematic figure illustrating double IVC with a retroaortic left renal vein, azygos continuation, and the presence of a hypoplastic hepatic portion of the IVC, drained into the right renal vein.

In fact in the same patient double IVC with a retroaortic left renal vein, azygos continuation of the IVC, and the presence of a hypoplastic hepatic segment of the IVC coexist the last one is drained into the right renal vein, at the confluence with right IVC, probably due to an incomplete atrophy of the right subcardinal vein (Figures 2 and 3). Therefore, with the partial atrophy of the prerenal division of the IVC, the blood returning from the lower extremities is partially shunted from the supracardinal system through the suprasubcardinal anastomosis to the retrocrural azygos vein, which is derived from the thoracic segment of the right supracardinal vein and partially shunted to the hepatic segment of the IVC through the residual prerenal segment.

Although the presence of combination of these venous variations in a patient is extremely rare, it should be recognized in order to eliminate the risk for severe hemorrhage during abdominal and thoracic surgeries or the risk for recurrent embolism during the placement of an IVC filter in vascular interventional procedures. In addition, awareness of the different anomalies of the IVC is necessary for radiologists to prevent misinterpretation of aberrant vessels as paravertebral lymph node enlargement and mediastinal masses.

Multidetector CT technique is the preferred method for imaging the congenital vascular anomalies of IVC since it is less costly, less invasive than conventional angiography, fast, easily applicable, and reliable in terms of identification of thoracoabdominal vascular structures. In fact MDCT imaging enables the acquisition of high-spatial-resolution volumetric image data during a single breath hold with the possibility of two-dimensional (2D) and three-dimensional (3D) image after processing, which allows the visualization of complex vascular malformations in an understandable way.
